# 2,4-Bis[bis(diisopropyl­amino)­phos­phanyl]-1,2,3,4-tetra­phospha­bicyclo[1.1.0]butane

**DOI:** 10.1107/S1600536808037938

**Published:** 2008-11-22

**Authors:** Agnieszka Łapczuk-Krygier, Katarzyna Baranowska, Jerzy Pikies

**Affiliations:** a Department of Inorganic Chemistry, Faculty of Chemistry, Gdańsk University of Technology, 11/12 G. Narutowicz St., 80952 - PL Gdańsk, Poland

## Abstract

The title compound, C_24_H_56_N_4_P_6_ or (^*i*^Pr_2_N)_2_P–P_4_–P(N^*i*^Pr_2_)_2_, adopts a butterfly structure, with planar environments for the N atoms and pyramidal environments for the P atoms. The structure studied has a 15% twin component that is related by a twofold rotation about [100].

## Related literature

For 2,4-bis­{[bis­(trimethyl­silyl)amido](diisopropylo­amido)­phosphido}1,2,3,4-tetra­phosphabicyclo­(1,1,0)butane, see: Bezombes *et al.* (2004 ▶). For 2,4-bis­(bis-di-*tert*-butyl­ophos­phido)­1,2,3,4-tetra­phosphabicyclo­(1,1,0)butane, see: Matern *et al.* (1997 ▶). For the handling of twinned diffraction data, see: Spek (2003 ▶).
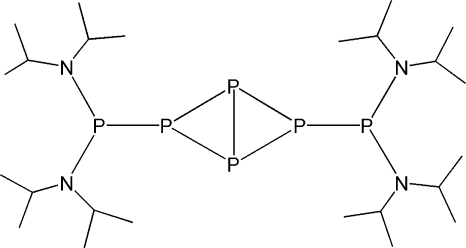


## Experimental

### 

#### Crystal data

C_24_H_56_N_4_P_6_
*M* *_r_* = 586.55
Monoclinic, 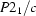

*a* = 13.3307 (5) Å
*b* = 20.9304 (7) Å
*c* = 12.8939 (5) Å
β = 109.349 (4)°
*V* = 3394.4 (2) Å^3^
*Z* = 4
Mo *K*α radiation
μ = 0.34 mm^−1^
*T* = 120 (2) K
0.46 × 0.14 × 0.06 mm

#### Data collection

Oxford Diffraction KM-4 CCD diffractometer
Absorption correction: analytical (*CrysAlis RED*; Oxford Diffraction, 2006 ▶) *T*_min_ = 0.954, *T*_max_ = 1.045
6310 measured reflections
6310 independent reflections
4727 reflections with *I* > 2σ(*I*)

#### Refinement

*R*[*F* ^2^ > 2σ(*F* ^2^)] = 0.050
*wR*(*F* ^2^) = 0.136
*S* = 1.04
6310 reflections
324 parameters
H-atom parameters constrained
Δρ_max_ = 0.61 e Å^−3^
Δρ_min_ = −0.51 e Å^−3^

### 

Data collection: *CrysAlis CCD* (Oxford Diffraction, 2006 ▶); cell refinement: *CrysAlis RED* (Oxford Diffraction, 2006 ▶); data reduction: *CrysAlis RED*; program(s) used to solve structure: *SHELXS97* (Sheldrick, 2008 ▶); program(s) used to refine structure: *SHELXL97* (Sheldrick, 2008 ▶); molecular graphics: *ORTEP-3 for Windows* (Farrugia, 1997 ▶); software used to prepare material for publication: *WinGX* (Farrugia, 1999 ▶).

## Supplementary Material

Crystal structure: contains datablocks I, global. DOI: 10.1107/S1600536808037938/ng2506sup1.cif

Structure factors: contains datablocks I. DOI: 10.1107/S1600536808037938/ng2506Isup2.hkl

Additional supplementary materials:  crystallographic information; 3D view; checkCIF report

## Figures and Tables

**Fig. 1.**
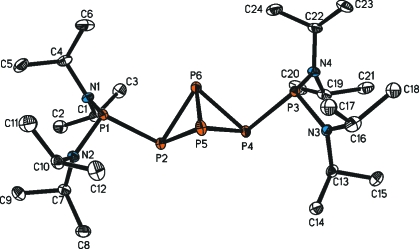
A view of the molecule of 1, showing the atom-labelling scheme. Displacement ellipsoids are drawn at the 30% probability level and H atoms have been omitted.

**Fig. 2.**
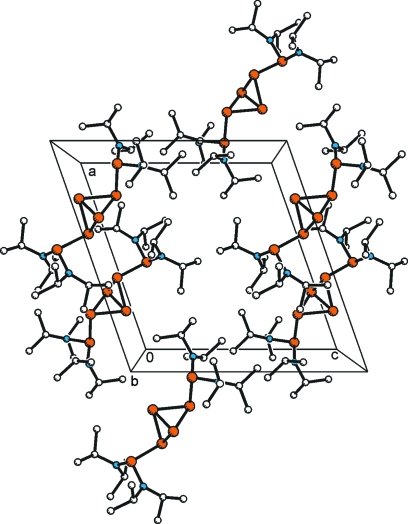
Packing diagram for 1. View direction is parallel to the crystallographic b axis.

## supplementary crystallographic information

### Comment

The molecular structure of 1 is shown in Fig.1. The crystal structure of 1 is build up of discrete molecules. The geometry of the phosphorous skeleton in 1 is similar to that in P_4_[(N^i^Pr_2_)(N(SiMe_3_)]_2_ (Bezombes *et al.*, 2004). The P1—P2 distance, 2.246 (2) Å, and P3—P4 distance, 2.243 (2) Å are slightly longer than P—P distances in the tetraphosphabicyclobutane core (P2,P5,P6,P4). The geometry around N atoms is strictly planar, the sum of angles around N2 atom is 360.8 °. The environments of P1 and P3 atoms are pyramidal, the sum of angles around P1 atom is 310.4 °. Clearly visible is very sharp pyramidal geometry around P2 and P4 atoms, the sum of angles around P2 atom is only 248.81 °.

### Experimental

All operations were carried out under purified nitrogen using Schlenk techniques. (^i^Pr_2_N)_2_PP(SiMe_3_)Li 2.5THF (0.170 g, 0.51 mmol) in toluene (5 ml) was dropped slowly to a suspension of Cp^*^ZrCl_3_(0.484 g, 0.94 mmol) in toluene (4 ml) at -30 °C.The reaction mixture turned immediately brown. The solution was allowed to stand one day at ambient temperature, then evaporated under reduced pressure and dried under vacuum (0.001 Torr) for 1 h. The residue was dissolved in 4 ml pentane and filtered. The solution was kept at 4 °C for two weeks and yielded small amount of colourless crystals of 1.

### Refinement

All C–H hydrogen atoms were refined as riding on carbon atoms with methyl C–H = 0.98 Å, methylene C–H = 0.99 Å and *U*_iso_(H)=1.2 *U*_eq_(C)for methylene CH and 1.5*U*_eq_(C) for methyl groups.

The structure initially refined to a rather high *R* index of 7.42%, the difference Fourier map showed relatively large peaks for an all-light atom structure (*ca* 1 e Å^-3 ^) and the weighting scheme used by the programme *SHELXL97* (Sheldrick, 2008) suggested unexpectedly large values for the second weighting parameters. A preliminary check with the TwinRotMat routine of *PLATON* (Spek, 2003) showed twofold twinning about [1 0 0]. Refinement against the TwinRotMat generated data gave a lower *R* index of 5.0%, final difference Fourier map (no peak larger than *ca* 0.6 e Å^-3^) and the second weight value decreased to 0.

### Crystal data

**Table:**

C_24_H_56_N_4_P_6_	*F*_000_ = 1272
*M**_r_* = 586.55	*D*_x_ = 1.148 Mg m^−^^3^
Monoclinic, *P*2_1_/*c*	Mo *K*α radiation λ = 0.71073 Å
Hall symbol: -P 2ybc	Cell parameters from 10425 reflections
*a* = 13.3307 (5) Å	θ = 2.5–32.4º
*b* = 20.9304 (7) Å	µ = 0.34 mm^−^^1^
*c* = 12.8939 (5) Å	*T* = 120 (2) K
β = 109.349 (4)º	Prism, colourless
*V* = 3394.4 (2) Å^3^	0.46 × 0.14 × 0.06 mm
*Z* = 4	

### Data collection

**Table:**

Oxford Diffraction KM-4 CCD diffractometer	6310 independent reflections
Monochromator: graphite	4727 reflections with *I* > 2σ(*I*)
Detector resolution: 8.1883 pixels mm^-1^	*R*_int_ = 0
*T* = 120(2) K	θ_max_ = 25.5º
ω scans, 0.75 deg width	θ_min_ = 2.5º
Absorption correction: analytical(CrysAlis RED; Oxford Diffraction, 2006)	*h* = −16→16
*T*_min_ = 0.955, *T*_max_ = 1.045	*k* = −25→25
6310 measured reflections	*l* = −13→15

### Refinement

**Table:**

Refinement on *F*^2^	Secondary atom site location: difference Fourier map
Least-squares matrix: full	Hydrogen site location: inferred from neighbouring sites
*R*[*F*^2^ > 2σ(*F*^2^)] = 0.050	H-atom parameters constrained
*wR*(*F*^2^) = 0.136	*w* = 1/[σ^2^(*F*_o_^2^) + (0.0841*P*)^2^] where *P* = (*F*_o_^2^ + 2*F*_c_^2^)/3
*S* = 1.04	(Δ/σ)_max_ < 0.001
6310 reflections	Δρ_max_ = 0.61 e Å^−^^3^
324 parameters	Δρ_min_ = −0.51 e Å^−^^3^
Primary atom site location: structure-invariant direct methods	Extinction correction: none

### Special details

**Table:**

Geometry. All e.s.d.'s (except the e.s.d. in the dihedral angle between two l.s. planes) are estimated using the full covariance matrix. The cell e.s.d.'s are taken into account individually in the estimation of e.s.d.'s in distances, angles and torsion angles; correlations between e.s.d.'s in cell parameters are only used when they are defined by crystal symmetry. An approximate (isotropic) treatment of cell e.s.d.'s is used for estimating e.s.d.'s involving l.s. planes.
Refinement. Refinement of *F*^2^ against ALL reflections. The weighted *R*-factor *wR* and goodness of fit *S* are based on *F*^2^, conventional *R*-factors *R* are based on *F*, with *F* set to zero for negative *F*^2^. The threshold expression of *F*^2^ > σ(*F*^2^) is used only for calculating *R*-factors(gt) *etc*. and is not relevant to the choice of reflections for refinement. *R*-factors based on *F*^2^ are statistically about twice as large as those based on *F*, and *R*- factors based on ALL data will be even larger.

### Fractional atomic coordinates and isotropic or equivalent isotropic displacement parameters (Å^2^)

**Table:**

	*x*	*y*	*z*	*U*_iso_*/*U*_eq_	
C1	−0.1377 (2)	0.37879 (15)	0.4006 (3)	0.0268 (7)	
H1	−0.0988	0.4188	0.4328	0.032*	
C2	−0.2520 (3)	0.39804 (17)	0.3362 (3)	0.0348 (8)	
H2A	−0.2524	0.426	0.2751	0.052*	
H2B	−0.2829	0.4207	0.3849	0.052*	
H2C	−0.2941	0.3597	0.3072	0.052*	
C3	−0.1350 (3)	0.33570 (18)	0.4960 (3)	0.0361 (8)	
H3A	−0.1759	0.2968	0.4679	0.054*	
H3B	−0.166	0.3582	0.5448	0.054*	
H3C	−0.0611	0.3243	0.5371	0.054*	
C4	−0.1270 (3)	0.28996 (15)	0.2755 (3)	0.0284 (7)	
H4	−0.1833	0.2767	0.3065	0.034*	
C5	−0.1821 (3)	0.29992 (18)	0.1524 (3)	0.0400 (9)	
H5A	−0.2318	0.3358	0.1407	0.06*	
H5B	−0.2212	0.2611	0.1201	0.06*	
H5C	−0.1288	0.3092	0.1174	0.06*	
C6	−0.0471 (3)	0.23567 (17)	0.2981 (4)	0.0459 (10)	
H6A	0.0082	0.2459	0.2662	0.069*	
H6B	−0.0834	0.1962	0.2651	0.069*	
H6C	−0.0146	0.2298	0.3777	0.069*	
C7	−0.0671 (2)	0.49548 (15)	0.2373 (3)	0.0254 (7)	
H7	−0.0924	0.4852	0.3002	0.03*	
C8	−0.0059 (3)	0.55820 (16)	0.2653 (3)	0.0357 (8)	
H8A	0.0566	0.5524	0.3312	0.053*	
H8B	−0.0519	0.5913	0.2791	0.053*	
H8C	0.017	0.5712	0.2037	0.053*	
C9	−0.1655 (3)	0.50184 (17)	0.1357 (3)	0.0323 (8)	
H9A	−0.1446	0.5165	0.0736	0.048*	
H9B	−0.2144	0.5329	0.15	0.048*	
H9C	−0.2009	0.4603	0.1181	0.048*	
C10	0.0504 (2)	0.44695 (16)	0.1414 (3)	0.0266 (7)	
H10	0.0203	0.4859	0.097	0.032*	
C11	0.0259 (3)	0.39069 (18)	0.0623 (3)	0.0414 (9)	
H11A	0.0546	0.3514	0.1027	0.062*	
H11B	0.0586	0.3978	0.0054	0.062*	
H11C	−0.0512	0.3865	0.0277	0.062*	
C12	0.1705 (3)	0.4575 (2)	0.1920 (3)	0.0414 (9)	
H12A	0.1842	0.4943	0.2418	0.062*	
H12B	0.2006	0.4657	0.1335	0.062*	
H12C	0.2037	0.4193	0.2331	0.062*	
C13	0.5113 (2)	0.46488 (15)	0.8092 (3)	0.0252 (7)	
H13	0.4396	0.4652	0.8182	0.03*	
C14	0.5113 (3)	0.51854 (16)	0.7295 (3)	0.0355 (8)	
H14A	0.5818	0.5217	0.7217	0.053*	
H14B	0.4938	0.559	0.7578	0.053*	
H14C	0.4582	0.5095	0.6577	0.053*	
C15	0.5919 (3)	0.47724 (17)	0.9223 (3)	0.0334 (8)	
H15A	0.5865	0.4436	0.973	0.05*	
H15B	0.5775	0.5188	0.9494	0.05*	
H15C	0.6637	0.4773	0.9173	0.05*	
C16	0.6260 (3)	0.39185 (17)	0.7457 (3)	0.0373 (9)	
H16	0.6652	0.4334	0.7631	0.045*	
C17	0.6113 (4)	0.3773 (3)	0.6272 (5)	0.090 (2)	
H17A	0.681	0.3745	0.6174	0.136*	
H17B	0.5696	0.4114	0.5805	0.136*	
H17C	0.5739	0.3365	0.6067	0.136*	
C18	0.6957 (3)	0.3420 (2)	0.8243 (6)	0.0786 (18)	
H18A	0.6628	0.2997	0.8065	0.118*	
H18B	0.7025	0.3531	0.9001	0.118*	
H18C	0.7663	0.3413	0.8163	0.118*	
C19	0.3926 (3)	0.34377 (15)	0.9308 (3)	0.0293 (8)	
H19	0.3947	0.3903	0.9141	0.035*	
C20	0.2820 (3)	0.32984 (19)	0.9340 (3)	0.0459 (10)	
H20A	0.2296	0.3379	0.8613	0.069*	
H20B	0.2672	0.3576	0.9884	0.069*	
H20C	0.2778	0.285	0.9542	0.069*	
C21	0.4770 (4)	0.33255 (18)	1.0423 (3)	0.0450 (10)	
H21A	0.4781	0.2872	1.0615	0.068*	
H21B	0.4605	0.3582	1.0981	0.068*	
H21C	0.5468	0.345	1.039	0.068*	
C22	0.4165 (3)	0.23797 (14)	0.8507 (3)	0.0277 (7)	
H22	0.4042	0.2273	0.9211	0.033*	
C23	0.5224 (3)	0.20904 (17)	0.8573 (3)	0.0386 (9)	
H23A	0.5361	0.2168	0.7882	0.058*	
H23B	0.5208	0.1629	0.8698	0.058*	
H23C	0.5789	0.2287	0.9182	0.058*	
C24	0.3262 (3)	0.20786 (17)	0.7590 (3)	0.0414 (9)	
H24A	0.2583	0.2255	0.7596	0.062*	
H24B	0.3266	0.1615	0.7699	0.062*	
H24C	0.3353	0.2171	0.6881	0.062*	
N1	−0.08069 (19)	0.35006 (11)	0.3314 (2)	0.0204 (6)	
N2	−0.00033 (19)	0.44163 (12)	0.2262 (2)	0.0206 (5)	
N3	0.52404 (19)	0.40146 (11)	0.7648 (2)	0.0212 (6)	
N4	0.4172 (2)	0.30869 (11)	0.8429 (2)	0.0216 (6)	
P1	0.03033 (6)	0.37788 (4)	0.31167 (6)	0.01830 (19)	
P2	0.10621 (6)	0.42397 (4)	0.47668 (6)	0.0214 (2)	
P3	0.43260 (6)	0.34204 (4)	0.72996 (6)	0.01890 (19)	
P4	0.28812 (6)	0.40449 (4)	0.66843 (6)	0.0212 (2)	
P5	0.27184 (6)	0.39792 (4)	0.49234 (7)	0.0271 (2)	
P6	0.18012 (7)	0.33459 (4)	0.55847 (7)	0.0273 (2)	

### Atomic displacement parameters (Å^2^)

**Table:**

	*U*^11^	*U*^22^	*U*^33^	*U*^12^	*U*^13^	*U*^23^
C1	0.0242 (17)	0.0277 (17)	0.0323 (18)	−0.0045 (14)	0.0146 (14)	−0.0053 (14)
C2	0.0264 (18)	0.0326 (19)	0.048 (2)	−0.0002 (14)	0.0157 (16)	−0.0008 (17)
C3	0.040 (2)	0.042 (2)	0.034 (2)	−0.0085 (16)	0.0225 (17)	−0.0024 (16)
C4	0.0321 (18)	0.0203 (16)	0.0359 (19)	−0.0082 (14)	0.0152 (15)	−0.0044 (14)
C5	0.045 (2)	0.035 (2)	0.035 (2)	−0.0159 (17)	0.0078 (17)	−0.0111 (16)
C6	0.049 (2)	0.0227 (19)	0.068 (3)	−0.0010 (16)	0.022 (2)	−0.0028 (18)
C7	0.0300 (18)	0.0221 (16)	0.0245 (17)	0.0012 (13)	0.0095 (14)	0.0031 (13)
C8	0.047 (2)	0.0247 (18)	0.036 (2)	−0.0013 (15)	0.0143 (17)	−0.0010 (15)
C9	0.0302 (19)	0.0337 (19)	0.0316 (19)	0.0067 (15)	0.0084 (15)	0.0076 (15)
C10	0.0279 (18)	0.0296 (18)	0.0240 (17)	0.0015 (14)	0.0110 (14)	0.0068 (14)
C11	0.060 (3)	0.042 (2)	0.031 (2)	−0.0044 (18)	0.0265 (18)	−0.0018 (17)
C12	0.032 (2)	0.058 (3)	0.037 (2)	−0.0070 (18)	0.0149 (17)	0.0092 (18)
C13	0.0190 (16)	0.0261 (17)	0.0309 (18)	−0.0033 (13)	0.0088 (13)	−0.0045 (14)
C14	0.0286 (19)	0.0298 (19)	0.043 (2)	−0.0019 (15)	0.0053 (16)	0.0037 (16)
C15	0.0345 (19)	0.036 (2)	0.0292 (18)	−0.0080 (15)	0.0100 (15)	−0.0089 (16)
C16	0.0242 (18)	0.037 (2)	0.056 (2)	−0.0031 (15)	0.0214 (17)	−0.0123 (18)
C17	0.072 (3)	0.140 (5)	0.092 (4)	−0.054 (3)	0.072 (3)	−0.068 (4)
C18	0.029 (2)	0.052 (3)	0.155 (6)	0.017 (2)	0.030 (3)	0.012 (3)
C19	0.049 (2)	0.0192 (16)	0.0272 (18)	0.0066 (15)	0.0228 (16)	0.0025 (13)
C20	0.060 (3)	0.042 (2)	0.052 (2)	0.0215 (19)	0.041 (2)	0.0212 (19)
C21	0.080 (3)	0.031 (2)	0.0234 (19)	−0.003 (2)	0.0167 (19)	−0.0030 (15)
C22	0.043 (2)	0.0166 (16)	0.0236 (17)	0.0040 (14)	0.0108 (14)	0.0033 (13)
C23	0.054 (2)	0.0259 (18)	0.037 (2)	0.0158 (16)	0.0170 (18)	0.0051 (16)
C24	0.061 (2)	0.0256 (19)	0.037 (2)	−0.0106 (17)	0.0155 (19)	−0.0005 (16)
N1	0.0239 (14)	0.0173 (13)	0.0229 (13)	−0.0054 (10)	0.0116 (11)	−0.0028 (10)
N2	0.0233 (13)	0.0207 (13)	0.0188 (13)	−0.0001 (10)	0.0082 (10)	0.0049 (10)
N3	0.0172 (13)	0.0207 (13)	0.0258 (14)	−0.0002 (10)	0.0074 (11)	−0.0030 (11)
N4	0.0292 (14)	0.0180 (13)	0.0193 (13)	0.0038 (11)	0.0105 (11)	−0.0003 (10)
P1	0.0177 (4)	0.0201 (4)	0.0168 (4)	0.0002 (3)	0.0052 (3)	0.0026 (3)
P2	0.0183 (4)	0.0241 (4)	0.0195 (4)	0.0007 (3)	0.0032 (3)	0.0009 (3)
P3	0.0197 (4)	0.0193 (4)	0.0169 (4)	0.0014 (3)	0.0050 (3)	−0.0009 (3)
P4	0.0187 (4)	0.0240 (4)	0.0191 (4)	0.0022 (3)	0.0037 (3)	0.0001 (3)
P5	0.0201 (4)	0.0415 (5)	0.0195 (4)	0.0020 (4)	0.0063 (3)	0.0047 (4)
P6	0.0255 (5)	0.0248 (4)	0.0251 (4)	−0.0041 (3)	−0.0003 (4)	0.0043 (4)

### Geometric parameters (Å, °)

**Table:**

C1—N1	1.479 (4)	C14—H14C	0.98
C1—C3	1.516 (5)	C15—H15A	0.98
C1—C2	1.529 (5)	C15—H15B	0.98
C1—H1	1	C15—H15C	0.98
C2—H2A	0.98	C16—N3	1.474 (4)
C2—H2B	0.98	C16—C17	1.505 (6)
C2—H2C	0.98	C16—C18	1.534 (6)
C3—H3A	0.98	C16—H16	1
C3—H3B	0.98	C17—H17A	0.98
C3—H3C	0.98	C17—H17B	0.98
C4—N1	1.479 (4)	C17—H17C	0.98
C4—C6	1.519 (5)	C18—H18A	0.98
C4—C5	1.526 (5)	C18—H18B	0.98
C4—H4	1	C18—H18C	0.98
C5—H5A	0.98	C19—N4	1.476 (4)
C5—H5B	0.98	C19—C20	1.516 (5)
C5—H5C	0.98	C19—C21	1.521 (5)
C6—H6A	0.98	C19—H19	1
C6—H6B	0.98	C20—H20A	0.98
C6—H6C	0.98	C20—H20B	0.98
C7—N2	1.472 (4)	C20—H20C	0.98
C7—C9	1.522 (4)	C21—H21A	0.98
C7—C8	1.525 (4)	C21—H21B	0.98
C7—H7	1	C21—H21C	0.98
C8—H8A	0.98	C22—N4	1.484 (4)
C8—H8B	0.98	C22—C23	1.512 (5)
C8—H8C	0.98	C22—C24	1.517 (5)
C9—H9A	0.98	C22—H22	1
C9—H9B	0.98	C23—H23A	0.98
C9—H9C	0.98	C23—H23B	0.98
C10—N2	1.469 (4)	C23—H23C	0.98
C10—C11	1.521 (5)	C24—H24A	0.98
C10—C12	1.531 (5)	C24—H24B	0.98
C10—H10	1	C24—H24C	0.98
C11—H11A	0.98	N1—P1	1.686 (2)
C11—H11B	0.98	N2—P1	1.692 (2)
C11—H11C	0.98	N3—P3	1.695 (2)
C12—H12A	0.98	N4—P3	1.688 (3)
C12—H12B	0.98	P1—P2	2.2482 (11)
C12—H12C	0.98	P2—P6	2.2121 (11)
C13—N3	1.477 (4)	P2—P5	2.2176 (11)
C13—C15	1.520 (4)	P3—P4	2.2438 (11)
C13—C14	1.522 (5)	P4—P6	2.2070 (11)
C13—H13	1	P4—P5	2.2123 (11)
C14—H14A	0.98	P5—P6	2.1610 (12)
C14—H14B	0.98		
			
N1—C1—C3	111.6 (3)	C13—C15—H15C	109.5
N1—C1—C2	113.5 (3)	H15A—C15—H15C	109.5
C3—C1—C2	110.5 (3)	H15B—C15—H15C	109.5
N1—C1—H1	107	N3—C16—C17	112.3 (3)
C3—C1—H1	107	N3—C16—C18	111.9 (3)
C2—C1—H1	107	C17—C16—C18	112.3 (4)
C1—C2—H2A	109.5	N3—C16—H16	106.6
C1—C2—H2B	109.5	C17—C16—H16	106.6
H2A—C2—H2B	109.5	C18—C16—H16	106.6
C1—C2—H2C	109.5	C16—C17—H17A	109.5
H2A—C2—H2C	109.5	C16—C17—H17B	109.5
H2B—C2—H2C	109.5	H17A—C17—H17B	109.5
C1—C3—H3A	109.5	C16—C17—H17C	109.5
C1—C3—H3B	109.5	H17A—C17—H17C	109.5
H3A—C3—H3B	109.5	H17B—C17—H17C	109.5
C1—C3—H3C	109.5	C16—C18—H18A	109.5
H3A—C3—H3C	109.5	C16—C18—H18B	109.5
H3B—C3—H3C	109.5	H18A—C18—H18B	109.5
N1—C4—C6	112.9 (3)	C16—C18—H18C	109.5
N1—C4—C5	111.7 (3)	H18A—C18—H18C	109.5
C6—C4—C5	111.6 (3)	H18B—C18—H18C	109.5
N1—C4—H4	106.7	N4—C19—C20	112.9 (3)
C6—C4—H4	106.7	N4—C19—C21	111.5 (3)
C5—C4—H4	106.7	C20—C19—C21	111.5 (3)
C4—C5—H5A	109.5	N4—C19—H19	106.8
C4—C5—H5B	109.5	C20—C19—H19	106.8
H5A—C5—H5B	109.5	C21—C19—H19	106.8
C4—C5—H5C	109.5	C19—C20—H20A	109.5
H5A—C5—H5C	109.5	C19—C20—H20B	109.5
H5B—C5—H5C	109.5	H20A—C20—H20B	109.5
C4—C6—H6A	109.5	C19—C20—H20C	109.5
C4—C6—H6B	109.5	H20A—C20—H20C	109.5
H6A—C6—H6B	109.5	H20B—C20—H20C	109.5
C4—C6—H6C	109.5	C19—C21—H21A	109.5
H6A—C6—H6C	109.5	C19—C21—H21B	109.5
H6B—C6—H6C	109.5	H21A—C21—H21B	109.5
N2—C7—C9	111.1 (3)	C19—C21—H21C	109.5
N2—C7—C8	112.9 (3)	H21A—C21—H21C	109.5
C9—C7—C8	111.7 (3)	H21B—C21—H21C	109.5
N2—C7—H7	106.9	N4—C22—C23	112.1 (3)
C9—C7—H7	106.9	N4—C22—C24	112.5 (3)
C8—C7—H7	106.9	C23—C22—C24	111.3 (3)
C7—C8—H8A	109.5	N4—C22—H22	106.8
C7—C8—H8B	109.5	C23—C22—H22	106.8
H8A—C8—H8B	109.5	C24—C22—H22	106.8
C7—C8—H8C	109.5	C22—C23—H23A	109.5
H8A—C8—H8C	109.5	C22—C23—H23B	109.5
H8B—C8—H8C	109.5	H23A—C23—H23B	109.5
C7—C9—H9A	109.5	C22—C23—H23C	109.5
C7—C9—H9B	109.5	H23A—C23—H23C	109.5
H9A—C9—H9B	109.5	H23B—C23—H23C	109.5
C7—C9—H9C	109.5	C22—C24—H24A	109.5
H9A—C9—H9C	109.5	C22—C24—H24B	109.5
H9B—C9—H9C	109.5	H24A—C24—H24B	109.5
N2—C10—C11	113.0 (3)	C22—C24—H24C	109.5
N2—C10—C12	111.6 (3)	H24A—C24—H24C	109.5
C11—C10—C12	111.1 (3)	H24B—C24—H24C	109.5
N2—C10—H10	106.9	C1—N1—C4	115.1 (2)
C11—C10—H10	106.9	C1—N1—P1	127.1 (2)
C12—C10—H10	106.9	C4—N1—P1	117.8 (2)
C10—C11—H11A	109.5	C10—N2—C7	117.2 (2)
C10—C11—H11B	109.5	C10—N2—P1	118.3 (2)
H11A—C11—H11B	109.5	C7—N2—P1	124.3 (2)
C10—C11—H11C	109.5	C16—N3—C13	114.7 (2)
H11A—C11—H11C	109.5	C16—N3—P3	118.6 (2)
H11B—C11—H11C	109.5	C13—N3—P3	126.7 (2)
C10—C12—H12A	109.5	C19—N4—C22	115.9 (2)
C10—C12—H12B	109.5	C19—N4—P3	125.3 (2)
H12A—C12—H12B	109.5	C22—N4—P3	118.4 (2)
C10—C12—H12C	109.5	N1—P1—N2	109.62 (12)
H12A—C12—H12C	109.5	N1—P1—P2	98.49 (9)
H12B—C12—H12C	109.5	N2—P1—P2	102.35 (9)
N3—C13—C15	112.9 (3)	P6—P2—P5	58.40 (4)
N3—C13—C14	112.1 (3)	P6—P2—P1	94.47 (4)
C15—C13—C14	111.3 (3)	P5—P2—P1	95.90 (4)
N3—C13—H13	106.7	N4—P3—N3	110.93 (13)
C15—C13—H13	106.7	N4—P3—P4	102.15 (9)
C14—C13—H13	106.7	N3—P3—P4	97.12 (9)
C13—C14—H14A	109.5	P6—P4—P5	58.55 (4)
C13—C14—H14B	109.5	P6—P4—P3	98.15 (4)
H14A—C14—H14B	109.5	P5—P4—P3	96.17 (4)
C13—C14—H14C	109.5	P6—P5—P4	60.60 (4)
H14A—C14—H14C	109.5	P6—P5—P2	60.68 (4)
H14B—C14—H14C	109.5	P4—P5—P2	80.46 (4)
C13—C15—H15A	109.5	P5—P6—P4	60.85 (4)
C13—C15—H15B	109.5	P5—P6—P2	60.93 (4)
H15A—C15—H15B	109.5	P4—P6—P2	80.70 (4)
			
C3—C1—N1—C4	63.7 (3)	C4—N1—P1—P2	−147.9 (2)
C2—C1—N1—C4	−62.0 (3)	C10—N2—P1—N1	−138.7 (2)
C3—C1—N1—P1	−115.5 (3)	C7—N2—P1—N1	47.2 (3)
C2—C1—N1—P1	118.9 (3)	C10—N2—P1—P2	117.5 (2)
C6—C4—N1—C1	−126.1 (3)	C7—N2—P1—P2	−56.6 (2)
C5—C4—N1—C1	107.2 (3)	N1—P1—P2—P6	84.13 (9)
C6—C4—N1—P1	53.1 (3)	N2—P1—P2—P6	−163.53 (9)
C5—C4—N1—P1	−73.6 (3)	N1—P1—P2—P5	142.77 (9)
C11—C10—N2—C7	−125.6 (3)	N2—P1—P2—P5	−104.88 (10)
C12—C10—N2—C7	108.4 (3)	C19—N4—P3—N3	53.6 (3)
C11—C10—N2—P1	59.9 (3)	C22—N4—P3—N3	−133.4 (2)
C12—C10—N2—P1	−66.2 (3)	C19—N4—P3—P4	−49.0 (3)
C9—C7—N2—C10	66.3 (3)	C22—N4—P3—P4	124.0 (2)
C8—C7—N2—C10	−60.1 (4)	C16—N3—P3—N4	111.4 (2)
C9—C7—N2—P1	−119.6 (3)	C13—N3—P3—N4	−71.5 (3)
C8—C7—N2—P1	114.1 (3)	C16—N3—P3—P4	−142.6 (2)
C17—C16—N3—C13	−120.2 (4)	C13—N3—P3—P4	34.5 (3)
C18—C16—N3—C13	112.5 (4)	N4—P3—P4—P6	−90.61 (10)
C17—C16—N3—P3	57.3 (4)	N3—P3—P4—P6	156.11 (10)
C18—C16—N3—P3	−70.1 (4)	N4—P3—P4—P5	−149.63 (10)
C15—C13—N3—C16	−63.2 (4)	N3—P3—P4—P5	97.09 (10)
C14—C13—N3—C16	63.5 (3)	P3—P4—P5—P6	95.80 (4)
C15—C13—N3—P3	119.6 (3)	P6—P4—P5—P2	61.62 (4)
C14—C13—N3—P3	−113.7 (3)	P3—P4—P5—P2	157.42 (4)
C20—C19—N4—C22	−61.8 (4)	P1—P2—P5—P6	−91.63 (4)
C21—C19—N4—C22	64.7 (4)	P6—P2—P5—P4	−61.54 (4)
C20—C19—N4—P3	111.4 (3)	P1—P2—P5—P4	−153.17 (4)
C21—C19—N4—P3	−122.2 (3)	P2—P5—P6—P4	−95.64 (4)
C23—C22—N4—C19	−121.2 (3)	P4—P5—P6—P2	95.64 (4)
C24—C22—N4—C19	112.5 (3)	P3—P4—P6—P5	−92.27 (4)
C23—C22—N4—P3	65.1 (3)	P5—P4—P6—P2	−61.81 (4)
C24—C22—N4—P3	−61.2 (3)	P3—P4—P6—P2	−154.08 (4)
C1—N1—P1—N2	−75.2 (3)	P1—P2—P6—P5	94.19 (4)
C4—N1—P1—N2	105.7 (2)	P5—P2—P6—P4	61.73 (4)
C1—N1—P1—P2	31.2 (3)	P1—P2—P6—P4	155.91 (4)
